# Dementia severity and weight loss: A comparison across eight cohorts. The 10/66 study

**DOI:** 10.1016/j.jalz.2012.11.014

**Published:** 2013-11

**Authors:** Emiliano Albanese, Clare Taylor, Mario Siervo, Robert Stewart, Martin J. Prince, Daisy Acosta

**Affiliations:** a Laboratory of Epidemiology, Demography, and Biometry, National Institute on Aging, National Institutes of Health, Bethesda, MD, USA; b Institute of Psychiatry, King's College London, London, UK; c Institute for Ageing and Health, Newcastle University, Newcastle on Tyne, UK; d Internal Medicine Department, Universidad Nacional Pedro Henriquez Ureña (UNPHU), Santo Domingo, Dominican Republic

**Keywords:** Dementia, Dementia severity, Weight loss, Developing countries, Epidemiology, Observational study, Older adults, Population-based sample, Cross-cohort, Meta-analysis, Aging

## Abstract

**Background:**

We aimed to investigate the association between dementia severity and weight loss in countries with low and middle incomes, where most prevalent cases reside.

**Methods:**

Cross-sectional catchment area surveys were performed in Cuba, Mexico, Venezuela, Peru, Dominican Republic, Puerto Rico, China, and India. In 16,538 older adults (≥65 years of age), significant weight loss was self-reported and confirmed by an informant. We conducted neuropsychological testing and clinical and neurological assessments. Dementia severity was determined by applying a validated algorithm and was quantified by the Clinical Dementia Rating Scale.

**Results:**

The characteristics of those who reported weight loss varied across countries. In Poisson models, after controlling for relevant covariates and for waist and arm circumferences, dementia severity was associated with reported weight loss (pooled prevalence ratios [95% confidence intervals {CI}] 2.19 [1.98, 2.41]; 3.81 [3.35, 4.33]; and 5.18 [4.41, 6.10] for CDR 0.5, 1, and 2/3, respectively, compared with CDR 0). The association increased linearly through stages of dementia severity in all countries (*P* for trend < .001), and between-country heterogeneity was minimal.

**Conclusions:**

We found a strong gradient effect in the direct association between dementia severity and reported weight loss, homogeneous across sites from eight countries, consistent with mechanistic data on the role of neurodegenerative processes on energy balance and with dietary changes due to disease severity. Considering the well-recognized effect of weight loss on morbidity and mortality and the large number affected by dementia in less resourced countries, amelioration of weight loss in dementia patients should be considered with priority in these settings.

## 1 Introduction

Weight loss in older adults is associated with increased disability and mortality risk [1]. Along with cancer and chronic diseases, dementia is a well-recognized source of weight loss.

Weight loss accrues with dementia severity and may in part be explained by reduced food intake because of impaired autonomy, eating disturbances, and reduced appetite [2]. However, mechanisms underlying weight loss in dementia remain unclear. Dementia neuropathology was inversely related to body mass index (BMI) in a recent postmortem study [3], and neurodegenenerative processes during the presymptomatic stage of the disease [4] may be responsible for involuntary weight loss [2] several years before the clinical onset of dementia [5], [6]. Epidemiologic evidence on the association between dementia and weight loss is lacking from low- and middle-income countries (LAMICs), where most people with dementia currently live [7] and where health-care systems and services are underresourced and less suited to the long-term needs of those affected by disabling chronic diseases [8].

The 10/66 study of aging and dementia is an epidemiologic investigation in which standardized procedures have been applied in identical surveys of over 17,000 older adults in LAMICs. The aim of the present analysis was to study the cross-sectional association between dementia severity, as quantified by the Clinical Dementia Rating Scale (CDR), and weight loss in each of these countries. We explored whether this association increased linearly through stages of dementia severity, and we formally measured heterogeneity across countries. Given the cross-sectional association between dementia severity and reduced lean mass [9], and because we have previously reported that dementia was cross-sectionally associated with low arm and waist circumferences in the 10/66 cohorts [10], we also considered their role in the association between dementia severity and weight loss.

## 2 Methods

### 2.1 Study design

The 10/66 surveys were performed using a one-phase design between January 2003 and July 2010 in urban and rural sites in Mexico, Peru, India, and China and in urban sites only in Cuba, Dominican Republic, Venezuela, and Puerto Rico. All older adults aged ≥65 years who resided in specified catchment areas were eligible with no exclusion criteria. Target sample sizes of 2000 per country were based on power calculations at 80% for an expected dementia prevalence of 4.5% (±0.9).

Details on the full 10/66 study protocol have been previously published [11]. In brief, measurements administered comprised a range of clinical and neurological assessments and structured questionnaires, including an informant interview, which were administered at the participants' homes by health workers or junior doctors (in Cuba and China), all of who received 1 week of training. Interviews and assessments followed a strict standard procedure, and the study protocol was translated into local languages. Local principal investigators conducted the studies under continuous supervision and with the assistance of the London coordinating center.

### 2.2 Weight loss

We defined weight loss of clinical significance in older people as >10 lb (4.5 kg), consistent with the geriatric syndrome of sarcopenia [12], and with studies on weight loss and mortality [13]. Weight loss was self-reported by participants, who answered the following question: “Have you lost more than 4.5 kg of body weight during the past 3 months?” For participants who were unable to quantify their weight loss with sufficient precision, interviewers explained that 10 lb/4.5 kg represents “significant weight loss.” The acceptability and appropriateness of this measure is supported by qualitative data obtained from the interviewers who recorded that weight loss was reported along with, for example, comments on “looser clothes” and “need to tighten the belt.” Moreover, reported weight loss was confirmed by a second source in the case of incapacity or cognitive impairment (this was the case for 2562 participants), and in the case of discrepancy, caregiver report was given salience.

### 2.3 Dementia diagnosis and severity

We used the 10/66 algorithm on the basis of information from the Community Screening Instrument for Dementia (CSI-D; composed of a participant cognitive examination and structured informant questionnaire regarding cognitive decline) [14], a modified version of the Consortium to Establish a Registry for Alzheimer's Disease (CERAD) 10-word recall task, and the Geriatric Mental State fully-structured clinical interview [15]. The 10/66 algorithm was previously developed and successfully validated in 26 countries [16], and its prospective validity has been confirmed [17]. Dementia severity (no dementia; questionable dementia; mild, moderate, and severe dementia) was determined by operationalizing the Washington University CDR criteria combining cognitive and functional information obtained from informants and patients, as detailed by Morris and colleagues [18].

### 2.4 Covariates

Age was established by consensus from the participant, informant, and documentation. Education was subdivided into five categories (no formal education, some formal education but less than primary, primary education completed, secondary education completed, and tertiary education completed). Socioeconomic status was measured by asking about household living circumstances (marital status and number of family members) and number of assets (motor vehicle, television, refrigerator and/or freezer, water, electricity, telephone, plumbed toilet/bathroom). Dietary habits were evaluated by asking participants the following questions: “How often do you eat meat/fish?” (4-point scale; never, some days, most days, or every day) and “How many portions of fruit and vegetables have you eaten in the last 3 days?” (number of portions reported). Self-reported physical activity was coded as very physically active, fairly physically active, not very physically active, and not at all physically active. Smoking status was coded as never, previous, or current. Hazardous drinking was defined as >21 units per week for men and >14 for women. Food insecurity (defined as any shortage of food that caused persistent hunger) and self-reported medical diagnoses of severe gastrointestinal disease, stroke, diabetes and ischemic heart disease were enquired about. Resting blood pressure was measured, and hypertension was defined as self-reported treatment or resting average systolic blood pressure ≥140 mmHg and/or average diastolic blood pressure ≥95 mmHg. Waist and arm circumferences were measured to the nearest centimeter (details have been previously reported) [10].

### 2.5 Statistical analysis

All analyses were performed on release 3.0 of the 10/66 data archive using Stata 11 (Stata Corp. College Station, TX) for men and women and urban/rural sites combined, adjusting for gender after having confirmed that the interaction terms of CDR with sex and urban/rural site on reported weight loss were not significant (*P* = .61 and .09, respectively). We combined the CDR “moderate” and “severe” dementia groups because of the paucity of severe dementia cases, and we used weight loss as a dichotomous dependent variable. We entered CDR as a categorical variable in Poisson models accounting for household clustering, and we calculated prevalence ratios (PRs) with robust 95% confidence intervals (CI) by country. Models were adjusted in stages for sociodemographic, lifestyle, and health characteristics, including reported preexisting vascular disease and dietary habits. In further adjusted models, we tested the robustness of these associations, also accounting for waist and arm circumferences, and we performed Sobel-Goodman mediation tests [7] to quantify the percentage of the total effect of dementia severity that was mediated by these anthropometric measures. We formally tested the existence of an incremental effect of dementia through stages of severity comparing PRs by CDR category using Wald tests by country. We measured between-country heterogeneity in the above associations using the Q-test and Higgins I^2^ obtained from fixed-effects meta-analytic models [19]. A fixed-effects model was chosen to make inferences about the common effect conditional on the 10/66 study centers. Random-effects models were not used because we did not aim to generalize to a hypothetical population of centers. We finally calculated pooled meta-analytic estimates for CDR 0.5, 1, and 2/3 compared with CDR 0 (i.e., no dementia), accounting for sample sizes and variances in country-specific estimates.

Ethical approval for the study was obtained from the Research Ethics Committee at King's College London and by local committees at each study site. Participants provided written informed consent: witnessed oral consent in the case of illiteracy or next-of-kin agreement in the case of incapacity or cognitive impairment.

## 3 Results

Response rates in all countries were high (>80%). The characteristics of participants (*n* = 16,538) with complete data are reported in Table 1. Weight loss prevalence ranged from 2% in China to 26% in the Dominican Republic, and it was lowest for participants with a CDR of 0 (i.e., no dementia) and highest in those with a CDR of 2/3 in all countries (Table 2). In the latter CDR category, the proportion (40.3%) of caregivers who reported weight loss discrepantly from participants was significantly higher (*P* = .002) compared with proportions in the less severe dementia categories (31.5% and 30.9% for CDR 0.5 and CDR 1, respectively).

As previously reported [20], life circumstances and sociodemographic, lifestyle, and health characteristics varied by country, but relatively little of this variation was associated with weight loss status (Table 1). Those reporting weight loss were older, more likely to be female (except in Mexico), had a lower educational level (except in China), had fewer household assets, were equally likely to live alone or with their spouse only (except in China and India), and were more or as likely to be smokers (except in Cuba and China). They reported eating fewer portions of meat, fish, and fruits or vegetables per week and had higher food insecurity compared with those who did not lose weight. Those who reported losing weight were less physically active and were more likely to have three or more physical impairments, including severe gastrointestinal disease and clinically diagnosed stroke, heart disease, and diabetes, and they had smaller arm and waist circumferences compared with those who did not report weight loss (Table 1). Correlations between reported weight loss and arm (−7.1%) and waist (−4.7%) circumferences were low.

**Fig. 1:**
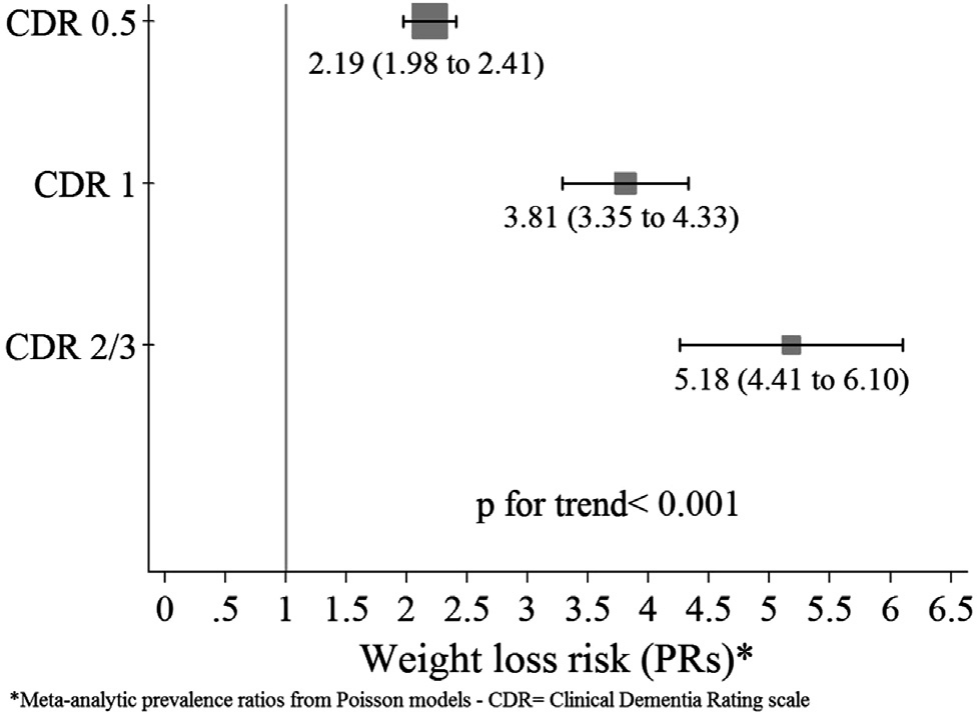
Increase in weight loss risk by dementia severity. Pooled estimates are from country-specific, fully adjusted Poisson models using fixed-effects method meta-analysis as for model 3 in Table 3 (i.e., adjusted for gender; age; education; number of assets; food insecurity [week frequency of hunger through shortage of food]; living circumstances [live alone or with spouse only vs larger families arrangements]; physical activity level [inactive, mild, moderate, high level]; smoking status [never, ex-, current smoker]; week meat and fish [none, some, or most days]; week portions of fruits and vegetables; number of physical impairments [≥3]; and reported clinical diagnosis of stroke, diabetes, and gastrointestinal and heart disease.

In unadjusted and fully adjusted models, dementia severity was positively associated with reported weight loss in all countries. Results were substantially unchanged after adjustments for waist and arm circumferences, although the mediation effects of arm and waist circumference were statistically significant (Sobel *P* < .001) with 1.4% and 0.5%, respectively, of the total effect (of CDR on reported weight loss) being mediated. In model 1, reported weight loss was incrementally associated with dementia severity such that those with mild dementia (CDR 1) were from three- (in Peru) to nearly eight-fold (in China) more likely to report weight loss compared with participants without dementia (Table 3). This incremental trend was significantly consistent across countries (all Wald tests for trend *P* values were <.001) (Table 3). Associations were only moderately attenuated when we controlled for sociodemographic, lifestyle, and life circumstances (model 2), and they were somewhat further attenuated when we additionally controlled for health characteristics (model 3) (Table 3). In fully adjusted models, the between-country heterogeneity was low or moderate (Table 3). The fixed-method meta-analytic pooled estimates confirmed the incremental trend in the association between dementia severity and reported weight loss observed at a country level (*P* < .001) (Fig. 1 and Figs. e-1–e-3).

## 4 Discussion

Dementia severity was independently associated with reported weight loss in >16,000 older adults (≥65) from eight countries with low and middle incomes. The association strengthened through stages of dementia severity, and it was not explained by covariates including low arm/waist circumferences and preexisting vascular disease. This association was highly consistent across countries, despite the high variability in weight loss and marked health and socioeconomic differences in the sample characteristics that would result in different confounding structures in each study site.

There are limitations in our study. Weight loss was self-reported; therefore, measurement error cannot be excluded and might have diluted our findings. This was because height and weight could not be feasibly measured in this large population-based study, which was performed in underresourced settings. The reliability of reported body weight and height has been reported to decrease with age [21]; however, cognitive status was unlikely to bias the information elicited in our study because this information was confirmed by an informant in all dubious cases (∼30% of participants with some cognitive impairment [CDR ≥ 0.5] who did not report weight loss). It is plausible that self-report would become less reliable at worse dementia severity, but this would result in measurement error and a bias toward the null. Our results confirmed this assumption, because >40% of informants reported weight loss in patients with more severe dementia (CDR 2/3) who did not report weight loss themselves. It is important to bear in mind that the catchment area sampling procedure cannot be assumed to provide nationally representative samples, and our findings should be generalized with caution. However, comparisons between country samples were appropriate, and internal validity was preserved through the standardized use of the 10/66 cross-culturally prevalidated research protocols [11]. Moreover, although CDR accuracy was probably lower than might be expected in clinical settings, all interviewers underwent intensive standardized 1-week training sessions and were closely supervised by local geriatricians and neurologists [11]. The cross-sectional design is another limitation of our study. Weight loss may occur during prodromal stages of dementia [6]; therefore, directionality in the observed association cannot be disentangled and reverse causality (i.e., weight loss causing more severe dementia) cannot be excluded. In fact, because impairment in activities of daily living (ADL) is part of the CDR construct, those with dementia who have another comorbid condition that may cause weight loss and impairment in ADL might be ranked with a more advanced CDR. Dementia and weight loss are recognized to increase mortality risk [22], [23]; therefore, survival bias might have occurred, although it most likely diluted rather than exaggerated true associations.

The geographic and cultural diversity of our sample is a major strength of our study, along with the very large sample size and the dementia algorithm, on the basis of measurements previously validated in over 20 countries [16]. However, given the size of the sample and settings, it was not possible to screen for nutritional deficiencies or determine *APOE* polymorphisms that might have confounded or modified the association between dementia severity and weight loss [24], and dementia subtypes were not considered in the present study. The magnitude of the associations remained marked in all countries, and it was largely unaffected after adjustment for a range of potential confounders, including life circumstances, dietary habits, and sociodemographic and health characteristics. Residual confounding cannot be excluded; however, the high homogeneity of our results across widely differing countries renders this less likely, which is additionally supported by the well-established evidence for an association between dementia and weight loss in Western settings.

Frail elders have poorer health and an increased mortality risk [25], and the association between dementia and weight loss has been consistently reported in clinical settings [26]. In prospective population-based studies, weight loss has been found to be associated with dementia even at mild severity, increasing with advancing disease severity and duration [27]. Our results are consistent with previous studies. Weight loss accelerated after, but also preceded, the symptomatic onset of dementia in samples of Japanese Americans [5], in Americans of Caucasian background [6], in Europeans [28], and in Americans of African origin [29] and Nigerians [30]. Similar findings have been reported in a prospective study of Australian men [31] and in a case-control study from Hong-Kong [32]. The homogeneity of our findings across world regions has not been previously reported. The present analysis extends previous findings in these samples of relatively consistent cross-sectional associations for dementia with smaller arm and waist circumference [10]; however, low arm and waist circumference only very marginally mediated the associations between reported weight loss and dementia severity. The novel homogeneity of our findings across very diverse settings, obtained using highly standardized methods, suggests that the association between dementia and weight loss may be relatively unaffected by ethnicity or geographical region.

Although this study did not seek to identify potential underlying mechanisms linking dementia with weight loss, its finding of relative homogeneity for this association in diverse populations should be taken into account when such mechanisms are being considered. Causal pathways may well vary during the disease course such that relative weight loss before the clinical onset of dementia and occurring afterward may have different underlying mechanisms [2]. For example, severity of dementia is accompanied by progressive functional impairment and worsening of behavioral and psychological symptoms as well as comorbid conditions that may result in reduced food intake, although these less obviously account for the weight loss that precedes the clinical onset. In Alzheimer's disease (which plausibly accounted for most dementia cases in our study), atrophy of the medial temporal lobe (a proxy for limbic system damage implicated in appetite control) and the hippocampal formation are early neuropathological features that might be implicated early on [33], [34]. Low BMI has also been found to be associated with Alzheimer pathology in a sample of older adults followed to postmortem [3]. Moreover, structural changes due to dementia may lead to hypothalamic dysfunction [35] and hypometabolism of the cingulate or hypothalamus [36], both of which may alter appetite control and energy metabolism [37]. Vascular pathways might also be implicated in any of these processes. In our study, adjustment for vascular disease and vascular risk factors had little effect on the association between dementia and weight loss in our analyses.

The sequelae of weight loss encompass increased mortality [23], morbidity, and worse prognosis in people with dementia [27]. Prevention and treatment of weight loss in dementia patients may improve the health status of the patients [2]. However, the effectiveness of nutritional interventions remains to be verified. Some interventions might be efficacious in dementia prevention [38], and treatment of weight loss is deemed critical in clinical settings and is highly recommended, particularly for institutionalized dementia patients [2]. The public health implications of weight loss prevention and treatment in dementia may be of particular relevance in LAMICs, where most of all dementia cases currently reside and where the steepest increases in numbers are also predicted [7]. Risk of malnutrition may be high, financial resources are scarce, and health systems are not designed for chronic diseases in these settings [39]. Evidence from further prospective observational and experimental studies would be helpful, and specific interventions should be designed and tailored to the significant cultural and resource diversities among non-Western countries. It is conceivable that simple and cost-effective recommendations to maintain or increase caloric intake of dementia patients may be predicted on precautionary principles of good clinical practice

**Research in context**

1. Systematic review: We searched Ovid and Medline databases for studies on weight loss and dementia since 1980. We combined free text and MeSH terms, including “Dementia,” “Cognitive Impairment,” (AND) “Weight loss” (OR) “epidemiology.” Relevant studies' quality was critically appraised considering study design and quality of reporting (on the basis of PRISMA recommendations).
2. Interpretation: In >17,000 older adults across diverse LAMICs, there was a strong gradient effect in the direct association between dementia severity and reported weight loss. In view of the effect of weight loss on morbidity and mortality, our results may have relevant public health implications.
3. Future directions: The association between dementia and weight loss should be studied prospectively, and body composition changes (i.e., in fat and lean mass) should be further investigated. The efficacy and effectiveness of nutritional interventions designed to ameliorate weight loss in dementia patients should be investigated, particularly in less resourced settings..

## Figures and Tables

**Table 1:** Characteristics of 10/66 study participants by country and by reported weight loss

	Weight loss	Cuba	Dominican Republic	Peru	Venezuela	Mexico	China	India	Puerto Rico
Response rate, %		94	95	84	80	85	85	85	92
Missing values, *n*		42	75	68	210	39	6	34	19
Sample size, *n* (%)	No	2669 (92)	1435 (74)	1499 (80)	1449 (83)	1723 (88)	2116 (98)	1679 (85)	1743 (88)
Yes	233 (8)	501 (26)	366 (20)	306 (17)	241 (12)	40 (2)	291 (15)	247 (12)
Age, mean (SD)	No	74.9 (7)	74.8 (7.3)	74.6 (7.3)	72.0 (6.6)	74.2 (6.6)	73.2 (6.1)	71.7 (5.9)	76.0 (7.1)
Yes	77.1 (7.5)	76.3 (7.8)	75.0 (7.1)	74.1 (7.4)	74.9 (6.6)	74.7 (6.4)	73.0 (6.1)	78.7 (8.6)
Females, *n* (%)	No	1717 (64)	931 (65)	912 (61)	896 (62)	1095 (64)	1188 (56)	945 (56)	1173 (67)
Yes	170 (73)	350 (70)	232 (63)	220 (72)	145 (60)	25 (63)	159 (55)	168 (68)
Less than primary education, *n* (%)	No	637 (24)	992 (69)	276 (18)	430 (30)	1210 (70)	1054 (50)	1259 (75)	382 (22)
Yes	80 (34)	381 (76)	71 (19)	104 (34)	176 (73)	19 (48)	244 (84)	75 (30)
Less than three assets, *n* (%)	No	67 (2.5)	197 (13.7)	69 (4.6)	21 (1.5)	369 (21.4)	112 (5.3)	831 (49.5)	3 (0.2)
Yes	9 (3.9)	97 (19.4)	24 (6.6)	13 (4.3)	54 (22.4)	1 (2.5)	206 (70.8)	1 (0.4)
Live alone or with spouse only, *n* (%)	No	640 (24.0)	295 (20.6)	212 (14.1)	157 (10.8)	462 (26.8)	692 (32.7)	327 (19.5)	1016 (58.3)
Yes	50 (21.5)	82 (16.4)	54 (14.7)	23 (7.5)	54 (22.4)	17 (42.5)	77 (26.5)	116 (47.0)
Current smoker, *n* (%)	No	523 (20)	173 (12)	52 (3)	166 (11)	155 (9)	490 (23)	544 (32)	85 (5)
Yes	36 (15)	67 (13)	14 (4)	34 (11)	22 (9)	7 (18)	122 (42)	16 (6)
Eat meat less than once a week, *n* (%)	No	90 (3)	74 (5)	98 (7)	259 (18)	141 (8)	56 (3)	313 (19)	101 (6)
Yes	10 (4)	33 (7)	49 (13)	76 (25)	31 (13)	3 (8)	56 (19)	14 (6)
Eat fish less than once a week, *n* (%)	No	246 (9)	473 (33)	124 (8)	57 (4)	488 (28)	60 (3)	346 (21)	401 (23)
Yes	38 (16)	178 (36)	27 (7)	19 (6)	65 (27)	5 (13)	67 (23)	72 (29)
Vegetables/fruits servings per week, mean (SD)	No	4.8 (3.6)	3.2 (3.2)	4.2 (2.8)	4.6 (3.5)	4.4 (3.6)	11.3 (4.8)	4.4 (2.5)	3.9 (2.3)
Yes	4.5 (3.4)	2.6 (2.9)	4.5 (3.0)	4.4 (3.6)	4.4 (3.6)	10.3 (3.7)	6.5 (2.8)	3.3 (2.4)
Have any food insecurity, *n* (%)	No	118 (4)	136 (9)	93 (6)	73 (5)	107 (6)	12 (1)	271 (16)	22 (1)
Yes	21 (9)	88 (18)	39 (11)	31 (10)	15 (6)	0 (0)	76 (26)	10 (4)
Not physically active, *n* (%)	No	823 (31)	467 (33)	410 (27)	449 (31)	576 (33)	1063 (50)	222 (13)	583 (33)
Yes	119 (51)	245 (49)	154 (42)	156 (51)	115 (48)	31 (78)	82 (28)	136 (55)
Physical impairments (≥3), *n* (%)	No	250 (9)	261 (18)	157 (10)	327 (23)	271 (16)	230 (11)	146 (9)	333 (19)
Yes	38 (16)	184 (37)	94 (26)	115 (38)	65 (27)	15 (38)	62 (21)	93 (38)
Have gastrointestinal problems, *n* (%)	No	221 (8)	239 (17)	170 (11)	256 (18)	248 (14)	73 (3)	58 (3)	317 (18)
Yes	32 (14)	132 (26)	89 (24)	77 (25)	49 (20)	6 (15)	15 (5)	74 (30)
Reported clinical diagnosis of stroke, *n* (%)	No	194 (7)	105 (7)	95 (6)	81 (6)	104 (6)	110 (5)	25 (1)	131 (8)
Yes	31 (13)	62 (12)	33 (9)	40 (13)	30 (12)	14 (35)	4 (1)	37 (15)
Reported clinical diagnosis of heart problems, *n* (%)	No	217 (8)	56 (4)	44 (3)	123 (8)	55 (3)	350 (17)	20 (1)	73 (4)
Yes	20 (9)	35 (7)	23 (6)	43 (14)	7 (3)	10 (25)	3 (1)	23 (9)
Waist circumference, mean (SD)	No	88.6 (13.2)	92.8 (12.8)	92.3 (9.6)	93.0 (11.5)	93.6 (10.7)	89.4 (11.3)	81.4 (10.6)	95.3 (13.3)
Yes	83.5 (12.5)	90.6 (12.9)	90.5 (10.3)	91.8 (12.2)	92.9 (12.4)	86.4 (9.5)	78.1 (11.3)	92.7 (14.4)
Arm circumference, mean (SD)	No	27.6 (5.3)	31.1 (6.9)	28.2 (4.3)	28.8 (7.9)	25.9 (4.4)	33.7 (5.5)	23.9 (4.0)	29.9 (5.1)
Yes	25.8 (4.8)	30.5 (6.9)	27 (4.8)	27.9 (4.7)	25.7 (4.9)	32.5 (6.2)	22.9 (3.4)	28.7 (4.9)
Reported clinical diagnosis of diabetes, *n* (%)	No	479 (18)	188 (13)	134 (9)	210 (14)	362 (21)	193 (9)	150 (9)	554 (32)
Yes	58 (25)	88 (18)	36 (10)	62 (20)	67 (28)	10 (25)	31 (11)	85 (34)

**Table 2:** Numbers of participants by dementia severity in each country and number (%) of those who lost weight by country and dementia severity

Country	Dementia severity							
CDR 0		CDR 0.5		CDR 1		CDR 2/3	
Total∗	Lost weight†	Total∗	Lost weight†	Total∗	Lost weight†	Total∗	Lost weight†
Cuba (*n* = 2902)	1655 (57.0)	75 (4.5)	967 (33.3)	88 (9.1)	161 (5.6)	40 (24.8)	119 (4.1)	30 (25.2)
Dominican Republic (*n* = 1936)	855 (44.2)	97 (11.4)	851 (44.0)	272 (32.0)	173 (8.9)	92 (53.2)	57 (2.9)	40 (70.2)
Peru (*n* = 1865)	1192 (63.9)	157 (13.2)	522 (28.0)	136 (26.1)	105 (5.6)	44 (41.9)	46 (2.5)	29 (63.0)
Venezuela (*n* = 1755)	923 (52.6)	93 (10.1)	734 (41.8)	166 (22.6)	76 (4.3)	32 (42.1)	22 (1.3)	15 (68.2)
Mexico (*n* = 1964)	918 (46.7)	52 (5.7)	913 (46.5)	142 (15.6)	113 (5.8)	38 (33.6)	20 (1.0)	9 (45.0)
China (*n* = 2156)	1380 (64.0)	9 (0.7)	659 (30.6)	16 (2.4)	77 (3.6)	4 (5.2)	40 (1.9)	11 (27.5)
India (*n* = 1970)	1076 (54.6)	62 (5.8)	785 (39.9)	187 (23.8)	93 (4.7)	34 (36.6)	16 (0.8)	8 (50.0)
Puerto Rico (*n* = 1990)	1104 (55.5)	64 (5.8)	695 (34.9)	103 (14.8)	131 (6.6)	49 (37.4)	60 (3.0)	31 (51.7)
All sample (*n* = 16,538)	9103 (55.0)	609 (6.7)	6126 (37.0)	1110 (18.1)	929 (5.6)	333 (35.8)	380 (2.3)	173 (4.5)

NOTE. Data are numbers (%).

∗ Denominators are total participants in each country.

† Denominators are participants in each dementia severity group by country.

**Table 3:** PR∗ and 95% CI for the association between dementia severity and weight loss compared with no dementia

Country	Dementia severity			
CDR 0.5	CDR 1	CDR 2/3	*P* value†
Model 1				
Cuba	2.01 (1.50–2.70)	5.45 (3.83–7.74)	5.33 (3.63–7.85)	<.001
Dominican Republic	2.79 (2.26–3.46)	4.67 (3.69–5.92)	6.14 (4.76–7.93)	<.001
Peru	1.97 (1.60–2.44)	3.18 (2.40–4.21)	4.77 (3.65–6.23)	<.001
Venezuela	2.18 (1.73–2.76)	4.06 (2.93–5.63)	6.45 (4.56–9.12)	<.001
Mexico	2.77 (2.04–3.75)	5.90 (4.07–8.55)	8.12 (4.69–14.06)	<.001
China	3.68 (1.63–8.32)	7.94 (2.49–25.38)	Not calculable	<.001
India	4.15 (3.16–5.46)	6.36 (4.44–9.10)	8.76 (5.10–15.05)	<.001
Puerto Rico	2.57 (1.91–3.45)	6.50 (4.70–8.98)	8.88 (6.26–12.58)	<.001
Model 2				
Cuba	1.89 (1.40–2.55)	4.30 (2.94–6.30)	3.53 (2.20–5.67)	<.001
Dominican Republic	2.57 (2.08–3.19)	4.19 (3.27–5.35)	5.81 (4.36–7.76)	<.001
Peru	1.88 (1.50–2.34)	3.11 (2.32–4.18)	4.67 (3.22–6.77)	<.001
Venezuela	2.05 (1.61–2.61)	3.27 (2.28–4.70)	4.12 (2.61–6.51)	<.001
Mexico	2.79 (2.05–3.80)	5.72 (3.83–8.56)	7.11 (3.67–13.75)	<.001
China	2.69 (1.16–6.24)	2.67 (0.79–8.98)	15.36 (5.96–39.57)	<.001
India	3.23 (2.43–4.31)	4.19 (2.85–6.14)	5.86 (2.90–11.87)	<.001
Puerto Rico	2.52 (1.86–3.40)	4.98 (3.43–7.24)	6.86 (4.57–10.31)	<.001
Model 3				
Cuba	1.87 (1.39–2.52)	4.29 (2.92–6.31)	3.45 (2.15–5.20)	<.001
Dominican Republic	2.41 (1.94–2.99)	4.01 (3.13–5.12)	5.46 (4.01–7.34)	<.001
Peru	1.64 (1.32–2.06)	2.92 (2.15–3.96)	4.66 (3.14–6.93)	<.001
Venezuela	1.96 (1.53–2.50)	2.86 (1.95–4.20)	4.05 (2.58–6.37)	<.001
Mexico	2.58 (1.89–3.52)	5.03 (3.31–7.65)	7.08 (3.56–14.09)	<.001
China	2.89 (1.18–7.07)	1.86 (0.40–8.78)	16.44 (5.62–48.06)	<.001
India	3.08 (2.31–4.11)	4.03 (2.73–5.95)	5.43 (2.57–11.46)	<.001
Puerto Rico	2.43 (1.80–3.27)	4.69 (3.21–6.84)	6.94 (4.57–10.53)	<.001
Meta-analysis‡				
Pooled estimate (95% CI)	2.19 (1.98–2.41)	3.81 (3.35–4.33)	5.18 (4.41–6.10)	<.001
Cochrane Q (*P* value)	Q = 16.393	Q = 9.350	Q = 11.929	
(7 degrees of freedom)	*P* = .022	*P* = .229	*P* = .103	
Higgins I^2^, % (95% CI)	57 (6–81)	25 (0–66)	41 (0–74)	

NOTE. Model 1 was adjusted for gender. Model 2 was adjusted for gender, age, education, number of assets, food insecurity (week frequency of hunger through shortage of food), living circumstances (live alone or with spouse only vs larger families arrangements), physical activity level (inactive, mild, moderate, high level), smoke status (never, ex-, current smoker), week meat and fish (none, some, or most days), and week portions of fruits and vegetables. Model 3 was additionally adjusted for number of physical impairments (≥3) and reported clinical diagnosis of stroke, diabetes, and gastrointestinal and heart disease.

∗ PRs are from Poisson models with robust 95% CI adjusted for household clustering.

† Wald test postestimations for linear hypothesis.

‡ Meta-analysis of country estimates from model 3.

## Supplementary data

Figs. e-1–e-3
