# Supplementary material for: Dementia severity and weight loss: A comparison across eight cohorts. The 10/66 study
Source: Alzheimers Dement. 2013 Nov;9(6):649–56. doi: 10.1016/j.jalz.2012.11.014 (PMC3898277; doi:10.1016/j.jalz.2012.11.014)
Supplement: Figs. e-1–e-3 [file mmc1.pdf]

Supplementary figures (3).

eFigure 1 Forrest plots of fixed-effects method meta-analysis of country-specific prevalence ratios (PRs) (95% CI), representing the association between probable dementia (CDR 0.5) and risk of weight loss, compared to no dementia.

eFigure 2 Forrest plots of fixed-effects method meta-analysis of country-specific prevalence ratios (PRs) (95% CI), representing the association between mild dementia (CDR 1) and risk of weight loss, compared to no dementia.

eFigure 3 Forrest plots of fixed-effects method meta-analysis of country-specific prevalence ratios (PRs) (95% CI), representing the association between moderate/severe dementia (CDR 2/3) and risk of weight loss, compared to no dementia. China is omitted due to wide CI.

eFigure 1

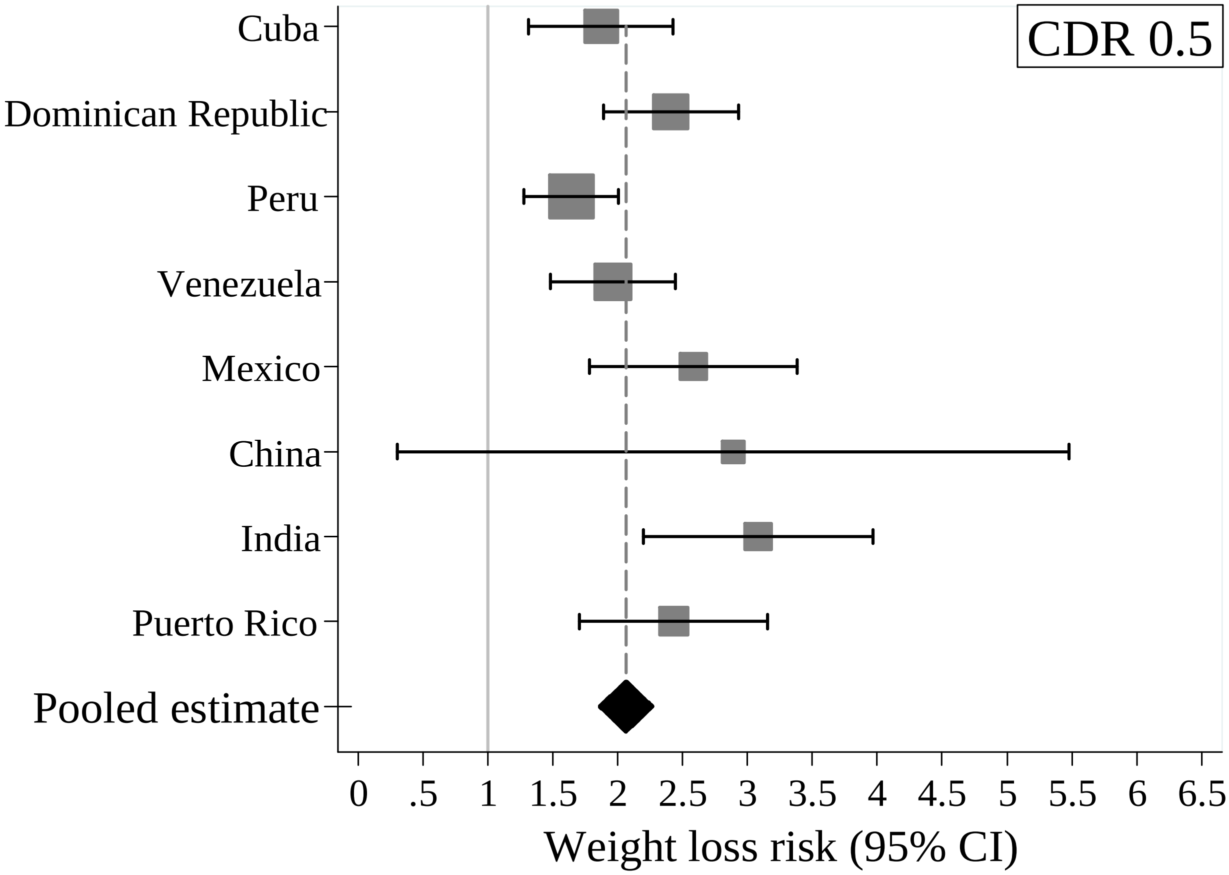

CDR 0.5
Cuba
Dominican Republic
Peru
Venezuela
Mexico
China
India
Puerto Rico
Pooled estimate
0 .5 1 1.5 2 2.5 3 3.5 4 4.5 5 5.5 6 6.5
Weight loss risk (95% CI)

eFigure 2

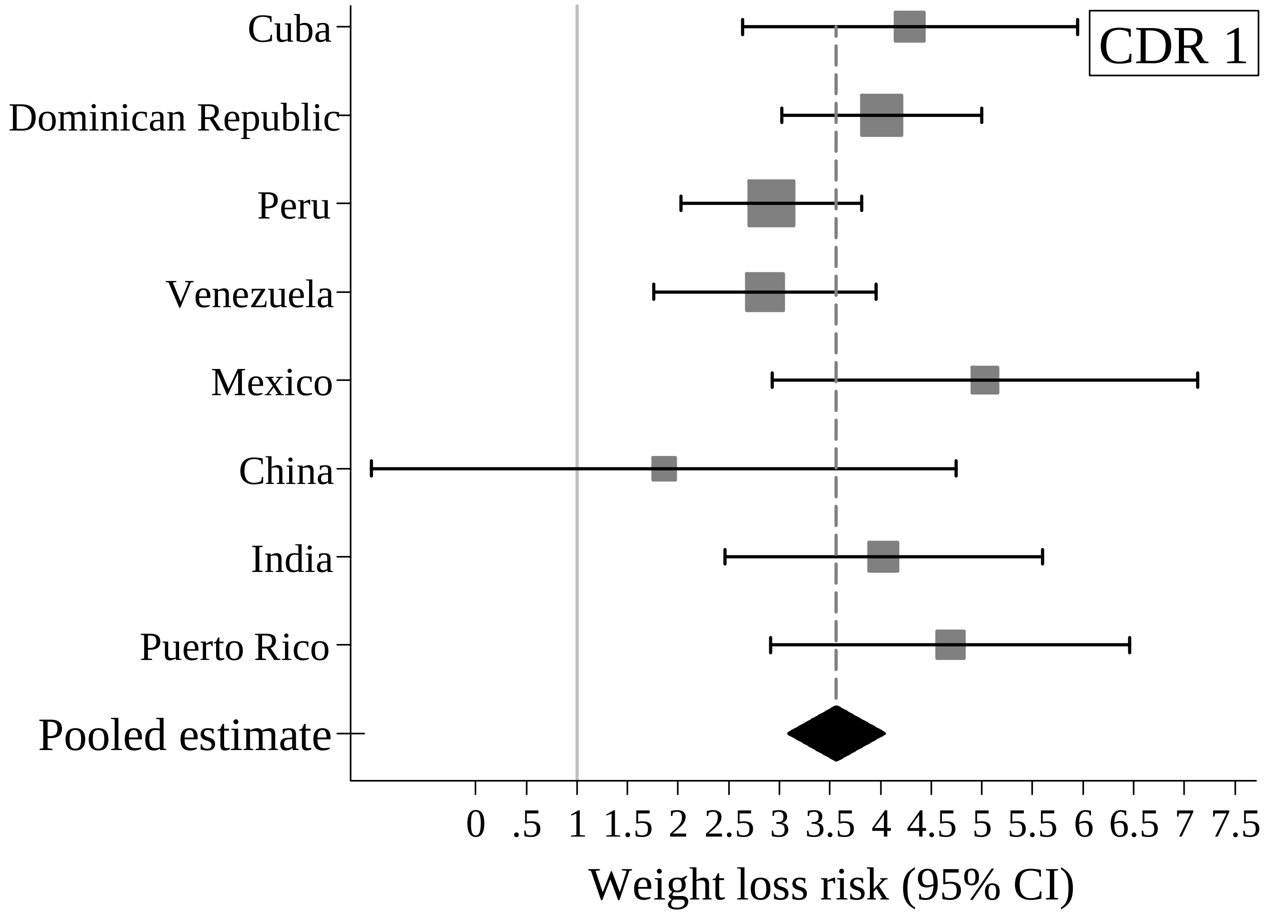
CDR 1
Cuba
Dominican Republic
Peru
Venezuela
Mexico
China
India
Puerto Rico
Pooled estimate
0 .5 1 1.5 2 2.5 3 3.5 4 4.5 5 5.5 6 6.5 7 7.5
Weight loss risk (95% CI)

eFigure 3

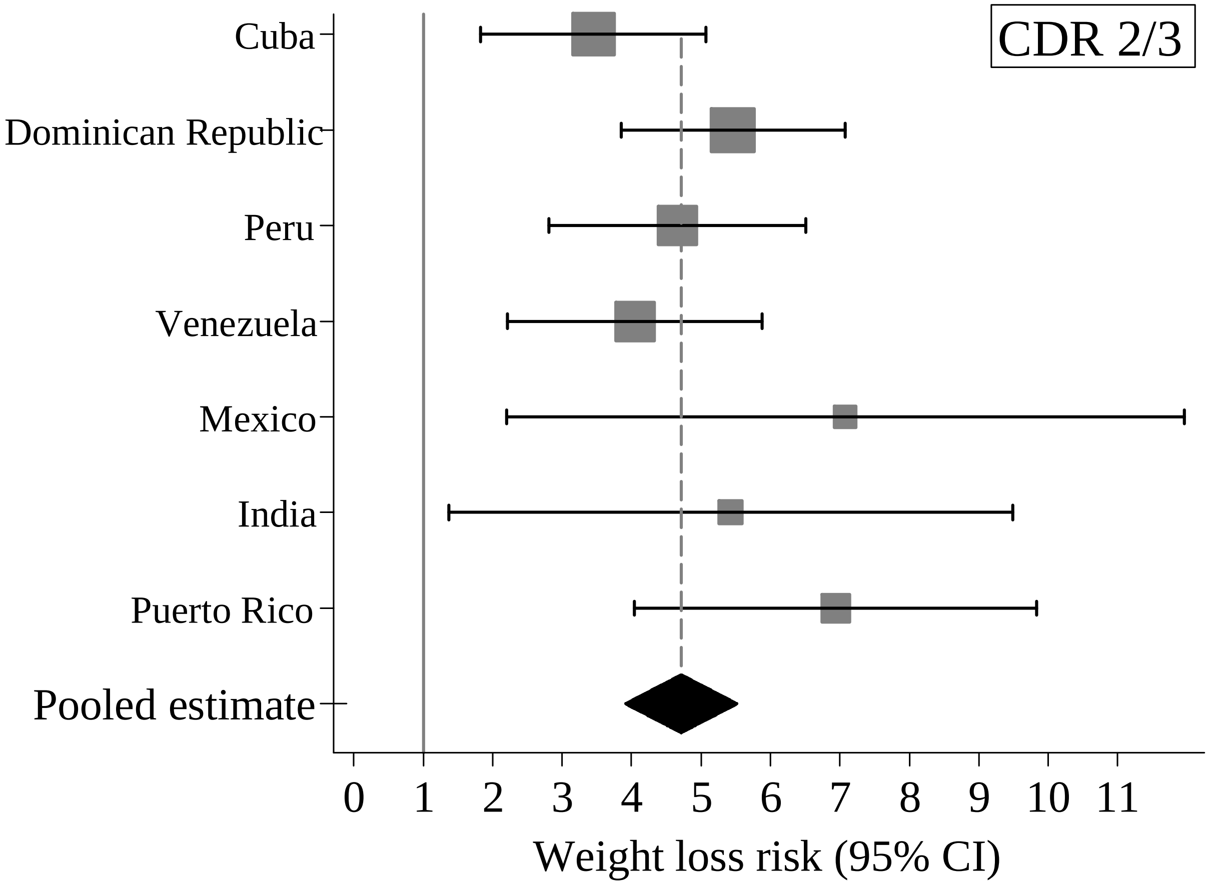
CDR 2/3
Cuba
Dominican Republic
Peru
Venezuela
Mexico
India
Puerto Rico
Pooled estimate
0 1 2 3 4 5 6 7 8 9 10 11
Weight loss risk (95% CI)
